# Spatiotemporal deep learning for early detection of isolated REM sleep behavior disorder and Parkinson’s disease using functional MRI data

**DOI:** 10.1038/s41531-026-01477-7

**Published:** 2026-07-14

**Authors:** Silvia Basaia, Stefano Pisano, Elisabetta Sarasso, Tania Filaferro, Andrea Gardoni, Sara Marelli, Roberta Balestrino, Tommaso Cusolito, Alessandra Castelnuovo, Andrea Grassi, Luigi Ferini-Strambi, Rosita De Micco, Alessandro Tessitore, Massimo Salvi, Filippo Molinari, Federica Agosta, Massimo Filippi

**Affiliations:** 1 Neuroimaging Research Unit, Division of Neuroscience, IRCCS San Raffaele Scientific Institute, Milan, Italy; 2 Neurotech Hub, Vita-Salute San Raffaele University, Milan, Italy; 3 Neurology Unit, IRCCS San Raffaele Scientific Institute, Milan, Italy; 4 Department of Neuroscience, Rehabilitation, Ophthalmology, Genetics and Maternal Child Health, University of Genoa, Genoa, Italy; 5 Biolab, PoliTo(BIO)Med Lab, Department of Electronics and Telecommunications, Politecnico di Torino, Torino, Italy; 6 Vita-Salute San Raffaele University, Milan, Italy; 7 Sleep Disorders Center, Division of Neuroscience, IRCCS San Raffaele Scientific Institute, Milan, Italy; 8 Neurorehabilitation Unit, IRCCS San Raffaele Scientific Institute, Milan, Italy; 9 Department of Advanced Medical and Surgical Sciences, University of Campania “Luigi Vanvitelli”, Napoli, Italy; 10 Neurophysiology Service, IRCCS San Raffaele Scientific Institute, Milan, Italy

**Keywords:** Biomarkers, Diseases, Neurology, Neuroscience

## Abstract

This study aimed to develop and evaluate a spatiotemporal deep-neural-network (stDNN) using resting-state fMRI (rs-fMRI) data to identify brain biomarkers associated with isolated REM sleep behavior disorder (iRBD) and Parkinson’s disease (PD) and to differentiate these conditions from controls. The final sample included 771 subjects, comprising 423 patients with PD, 144 with iRBD, and 204 healthy controls. stDNN model was applied to mean timeseries extracted for each subject from rs-fMRI data. By integrating spatio-temporal features, the network classified subjects based on distinct neural patterns. Model generalizability was assessed using subject-wise k-fold cross-validation. Explainable artificial intelligence (XAI) methods were applied. stDNN achieved balanced accuracy rates of 71.0% in distinguishing controls from PD and up to 71.9% in middle-stage PD cases. It also demonstrated over 80% accuracy in differentiating healthy controls from iRBD. XAI analysis highlighted the involvement of fronto-parietal and temporal regions, including the dorsolateral prefrontal cortex, and anterior temporal gyri, in distinguishing controls from PD. In the comparison with iRBD, key contributing areas included the bilateral superior and medial frontal gyri, dorsolateral prefrontal cortex, parietal and occipital regions (lingual gyri and cuneus). This study demonstrates the potential of stDNN to differentiate between iRBD, PD, and controls using rs-fMRI data.

## Introduction

Isolated REM sleep behavior disorder (iRBD) is recognized as one of the strongest prodromal biomarkers of Parkinson’s disease (PD), with longitudinal studies showing a conversion risk exceeding 80% over the long term^1–3^. iRBD thus represents an ideal model for the early investigation of neurofunctional changes that precede motor onset, particularly since structural MRI has limitations in detecting prodromal PD due to the absence of significant structural changes in its initial stages^4–6^. In contrast, resting-state functional MRI (rs-fMRI) has shown promise in identifying early alterations in brain functional connectivity, which may precede observable structural damage^7–10^. However, the application of rs-fMRI to single-subject analyses remains constrained by traditional statistical and analytical methods, which often rely on group-level inferences and lack the sensitivity required for individual diagnosis. This limitation has resulted in rs-fMRI being primarily utilized in research settings rather than in clinical practice.

Rs-fMRI signal analysis remains methodologically complex: it is a non-stationary signal, sensitive to both physiological and technical noise, and highly variable across individuals and sessions^11^. Traditionally, researchers have relied on aggregated measures such as static functional connectivity, regional homogeneity, or amplitude of low-frequency fluctuations, but these metrics tend to oversimplify the temporal richness of the signal^12^.

In recent years, data-driven deep learning models have begun to be successfully applied to rs-fMRI data. Architectures based on 1D convolutions over atlas-parcellated time series have shown a strong ability to capture meaningful temporal features while preserving spatial relationships and maintaining a manageable number of parameters^13,14^, later evolving into more sophisticated spatiotemporal models^15^. The spatiotemporal Deep Neural Network (stDNN) architecture, first introduced by Ryali et al.^16^, employs multiple 1D convolutional blocks to expand the temporal receptive field and model complex temporal dependencies directly from fMRI time series, followed by temporal averaging to integrate information across time. This approach provided high accuracy (>90%) in differentiating sex differences from rs-fMRI timeseries across multiple sessions and three independent cohorts, highlighting its suitability for end-to-end modeling while avoiding traditional feature engineering. In the present study, the same architecture was adopted as the starting point for our study and further refined to address the specific context of our application.

These recent advances in deep learning approaches offer new opportunities for developing reliable biomarkers for early disease detection. Following this paradigm, the present study applies an stDNN to a multicenter dataset including subjects with iRBD, PD, and healthy controls, with the aim of: 1) identifying distinct functional patterns for each group; 2) assessing the model’s effectiveness in distinguishing iRBD from healthy controls as a potential early biomarker; and 3) exploring the hypothesis of neurofunctional continuity between iRBD and PD.

## Results

### stDNN classification

A summary of the model’s cross-validated performances across the different classification tasks are reported in Table 1 and Supplementary Table 1.

**Table 1:** Demographic and clinical features of controls, iRBD and PD patients in all cohorts

	Controls	PD	iRBD
OSR cohort			
*N*	129	86	44
Age [years]	65.17 ± 8.06 (41–84)	66.12 ± 7.79 (46–82)	65.46 ± 7.19 (51–80)
Sex [women/men]	63/66	58/28^a^	36/8^a^
Disease duration [years]	–	6.59 ± 3.88 (0.5–16.0)	6.54 ± 3.08 (5.0–20.0)
UPDRS III	–	33.21 ± 9.57 (13–62)	*XXX*
Hoehn & Yahr	–	2.24 ± 0.40 (1–3)	–
**Naples cohort**			
*N*	38	90	/
Age [years]	60.24 ± 5.67 (50–69)	62.92 ± 1.01 (41–83)	/
Sex [women/men]	18/20	47/43	/
Disease duration [years]	–	1.27 ± 0.66 (0.3–3.0)	/
UPDRS III	–	20.02 ± 8.82 (5–46)	/
Hoehn & Yahr	–	1.54 ± 0.52 (1–2.5)	/
**PPMI cohort**			
*N*	37	247	100
Age [years]	65.31 ± 11.23 (32–85)	64.16 ± 9.68 (34–84)	66.84 ± 6.48^b^ (51–82)
Sex [women/men]	28/9	157/90	87/13^b^
Disease duration [years]	–	1.08 ± 1.19 (0.1–12.2)	3.20 ± 5.00 (0.2–30.0)
UPDRS III	–	23.87 ± 10.58 (3–56)	*XXX*
Hoehn & Yahr	–	1.73 ± 0.46 (1–3)	–

Values are reported as means ± standard deviations (min–max). The threshold of statistical significance was set at *p* < 0.05. *p* values refer to ANOVA models followed by post-hoc, Bonferroni-corrected comparisons or Pearson’s chi square, as appropriate.

*iRBD* isolated REM sleep behavior disorder, *PD* Parkinson’s disease.

^a^Statistically significant difference with controls.

^b^Statistically significant difference with PD.

*Controls vs PD*. The primary classification task aimed to distinguish PD patients from healthy controls. The model achieved a balanced accuracy of 71.0% (84.1% sensitivity, 57.8% specificity). The long short-term memory (LSTM) baseline model performed significantly worse, with balanced accuracy not exceeding 50%. Stratifying PD patients by disease severity (early-stage *vs* controls and middle-stage *vs* controls) did not improve overall accuracy but led to increased specificity at the cost of reduced sensitivity (the ability to effectively classify PD subjects as affected). The classification of early-stage PD versus middle-stage PD achieved a balanced accuracy of 68.3% (69.3% sensitivity, 67.4% specificity), with similar values for the other metrics. A detailed summary of the classification performance obtained with the different UPDRS-III thresholds is provided in Supplementary Table 1.

*Controls vs iRBD*. The model achieved a balanced accuracy of 71.1% in distinguishing iRBD patients from controls. Applying transfer learning significantly improved performance, increasing balanced accuracy to 82.1%. The model also achieved F1 score, specificity, and precision above 80%, while sensitivity reached 75%.

*iRBD vs PD*. The classification between iRBD and PD proved challenging, with balanced accuracy reaching only 52.0% (73.5% sensitivity, 30.5% specificity) in cross-validation. Transfer learning provided modest improvement, with accuracy remaining below 60%. This performance did not improve significantly when comparing iRBD against advanced-stge PD cases only.

### Model explainability

**Fig. 1:**
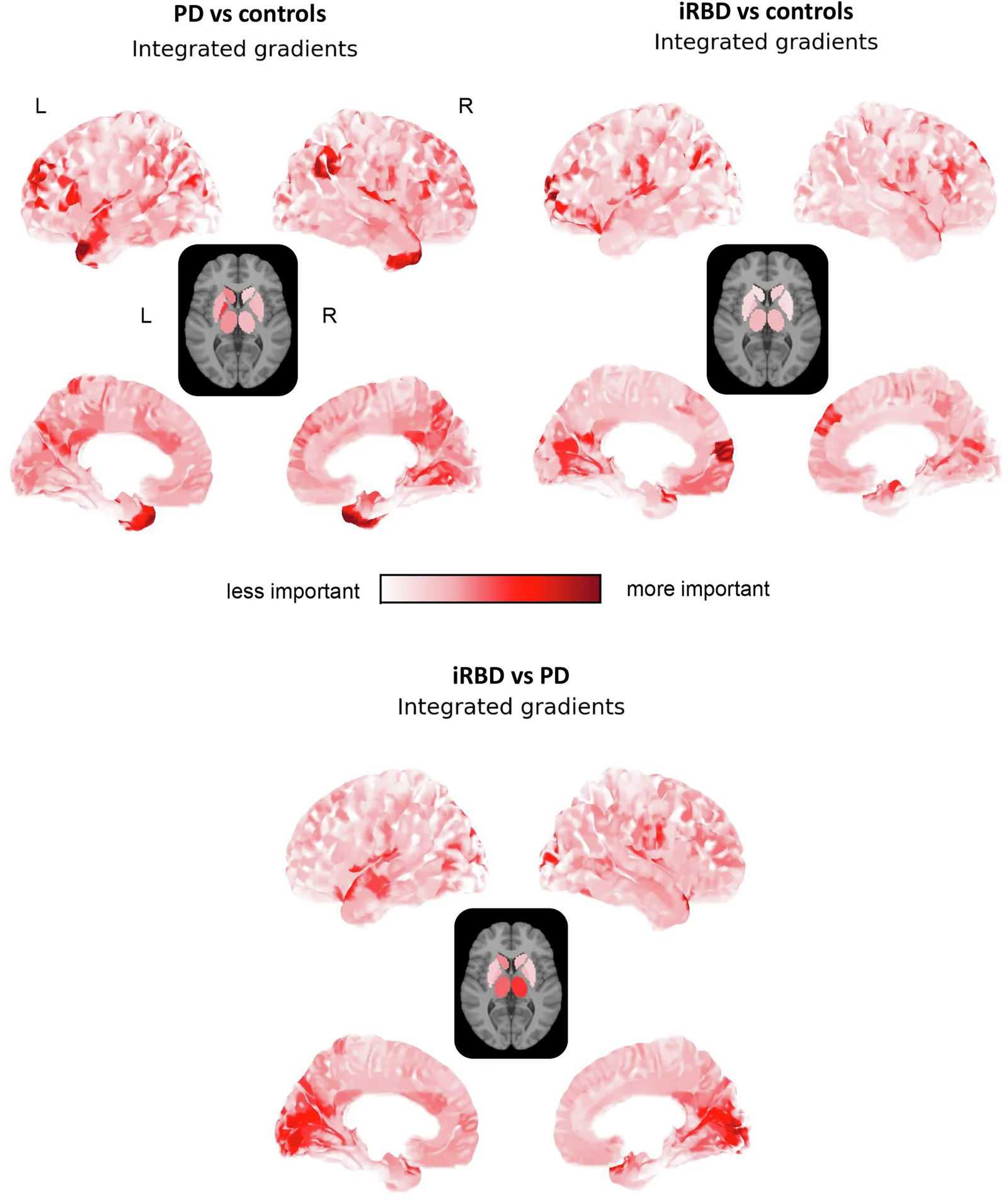
Group-level brain fingerprints from integrated gradients. Group-level integrated gradients attribution maps derived from the stDNN for the pairwise comparisons PD vs controls, iRBD vs controls, and iRBD vs PD. Group-level fingerprints are shown, computed as the average of individual IG fingerprints across all participants within each group, to emphasize the brain regions that contribute most consistently to the classification. For each comparison, cortical regions are shown for the left and right hemispheres, together with a representative axial slice with subcortical structures. Color intensity reflects the contribution of each region to the model, ranging from white for lower values and red for higher values. iRBD isolated REM sleep behavior disorder, L left, PD Parkinson’s disease, R right.

Figure 1 illustrates the key regions identified through IG analysis for the binary classifications. For the controls *vs* PD classification, the most relevant MRI features for accurate classification were located in the frontal, parietal, temporal and occipital regions. Specifically, the regions contributing most to the classification included the bilateral dorsolateral prefrontal cortex, with a left hemispheric predominance, and the bilateral precentral gyri within the frontal lobe; the anterior portions of the superior and middle temporal gyri bilaterally within the temporal lobe; the inferior parietal lobule, supramarginal gyri, and precuneus within the parietal lobe; and the bilateral lingual gyri, with a right hemispheric predominance, within the occipital lobe.

For the classification between healthy controls *vs* iRBD patients, the regions contributing most to the model included the bilateral superior frontal gyrus and anterior medial frontal gyri, as well as the dorsolateral prefrontal cortex with a left hemispheric predominance, within the frontal lobe; parietal regions encompassing the bilateral postcentral gyri, the left inferior parietal gyrus, and the left precuneus; and occipital regions bilaterally, including the lingual gyri and cuneus.

For the iRBD *vs* PD classification, the interpretation of IG results should be approached with caution due to the model’s relatively low accuracy. Within these limitations, the analysis primarily highlighted medial occipital regions as the main contributors to classification performance, particularly the bilateral lingual gyri and cuneus, as well as the precuneus and the left superior temporal gyrus.

## Discussion

In the present study, we applied a recently introduced deep learning architecture to classify patients with iRBD, PD and healthy controls using a multi-centre dataset. The deep learning model demonstrated relatively high accuracy in distinguishing between the three groups in binary classification tasks. Our findings demonstrate that functional brain dynamics, obtained without relying on connectivity features, contain disease-relevant information that can be effectively captured by deep learning models. The model achieved good accuracies (71%) in both controls vs PD and controls vs iRBD classifications. Notably, performance in the iRBD classification task improved 82.1% through transfer learning. These findings highlight the model’s capacity to generalize across tasks and suggest potential clinical relevance, pending external validation, for early-stage diagnosis and differential classification.

The neural network proposed in this study represents a significant advancement in the classification of neurological subjects based on rs-fMRI signals. Unlike traditional methods that rely on aggregated measures such as static functional connectivity, the stDNN architecture is specifically designed to capture the temporal and spatial complexity of the BOLD signal without restrictive assumptions of stationarity. This capability comes from an architecture composed of multiple 1D convolutions applied along the temporal dimension of the parcellated time series, followed by a temporal pooling phase. This allows the model to learn compact yet meaningful representations of the dynamic signal. Building upon this foundation, the stDNN architecture^16^ improves over other DNN architectures by effectively modeling both spatial and temporal correlations in 4D fMRI data. Traditional methods, such as 2D and 3D CNNs for spatial data, and 1D CNNs, LSTMs, or transformers for temporal data, struggle to efficiently capture the complex spatiotemporal relationships in fMRI due to issues like a large number of parameters^17^ and the difficulty in training with limited labeled data, a common issue in the medical field, which can lead to overfitting^18^.

A further significant aspect of this study is the incorporation of explainability techniques. Traditional deep learning models, especially those applied to time-series data such as rs-fMRI, are often viewed as “black-box” models, with little transparency into which features drive the predictions. To address this limitation, an Explainable AI (XAI) approach was incorporated to interpret the stDNN model’s decisions. This approach allowed us to identify the specific brain regions and connectivity patterns that predominantly contributed to the differentiation between the groups. This interpretability is crucial for ensuring that the model’s findings are not only accurate but also biologically meaningful and clinically applicable.

Another clear strength emerging from the results is the substantial outperformance of LSTM-based models. Despite the popularity of LSTMs for time series analysis, in this context the model showed unsatisfactory performance, with a balanced accuracy below 50% in the control *vs* PD classification task. This suggests that LSTMs are not well suited for capturing the distributed and simultaneous brain dynamics characteristic of fMRI signals. In contrast, the stDNN achieved a balanced accuracy of 71% on the same task, demonstrating a higher generalization capability and greater sensitivity to the complex functional alterations typical of PD. This improvement reflects the greater parameter efficiency of the stDNN, which, thanks to the compact structure of 1D convolutions, avoids overfitting even in the presence of limited labeled data, a common condition in clinical neuroimaging.

The model successfully distinguished PD patients from controls, achieving a balanced accuracy of 71%, suggesting that it captured discriminative functional patterns between groups. From a methodological perspective, this performance is broadly comparable to previous rs-fMRI–based machine learning studies in PD, which reported accuracies ranging from approximately 67% in external validation settings to about 76–81% in primary or single-center cohorts. Importantly, the model also showed encouraging generalizability within the available multicenter dataset^19,20^. For the controls vs PD classification, the most relevant regions included frontal, parietal, temporal, and occipital cortices, supporting the notion that PD-related functional alterations extend beyond motor circuits and involve large-scale associative networks. In particular, the contribution of the dorsolateral prefrontal cortex, inferior parietal lobule, and precuneus is consistent with previous evidence of fronto-parietal and default-mode network dysfunction in PD, while precentral, temporal, and lingual regions reflect the involvement of sensorimotor and posterior cortical systems. Overall, these findings indicate that the stDNN captured biologically plausible functional patterns associated with PD^21,22^. Together, these findings suggest that the stDNN captured biologically plausible functional patterns in PD.

One of the most significant findings of the study is the promising performance of the stDNN model in distinguishing subjects with iRBD from healthy controls. Although the model already achieved promising results with its standard architecture, it was the application of transfer learning that led to a substantial qualitative leap: by using a model pre-trained on the PD *vs* controls task and subsequently fine-tuned for the iRBD *vs* controls task, a balanced accuracy of 82.1% was achieved, with high values for specificity, precision, and F1-score (all exceeding 80%). The performance improvement due to transfer learning demonstrates that the functional alterations present in iRBD are already learned during the PD training phase, reinforcing the notion of a pathophysiological continuum between the two conditions^23–26^. This result suggests that the model was able to identify functional patterns common to both PD and iRBD, consistent with the hypothesis that iRBD represents a prodromal stage of PD^2,27^. In the existing literature, there is limited research on machine learning applied to rs-fMRI in the context of iRBD; a notable exception is the study conducted by Matsushima et al.^25^ which used functional‑connectivity features to classify individuals with iRBD relative to healthy controls and reported only moderate performance, with accuracy values in the range of 65–70% across different evaluation metrics. The explainability analysis based on integrated gradients enabled the identification of the most relevant brain regions for the classification task. Notably, the precuneus, the left dorsolateral prefrontal cortex, the superior and medial frontal gyri, the left superior temporal gyrus, and the occipital gyrus stood out. These regions, previously implicated in studies on iRBD and risk of conversion to PD^4,28^, reflect early dysfunction of fronto-occipital circuits, which are responsible for executive cognitive functions, complex visual processing, and self-awareness. These findings lay the groundwork for using the stDNN as a potential candidate biomarker, although further validation is needed before any clinical translation. Its accuracy in detecting early functional alterations suggests a possible clinical application in risk stratification among iRBD subjects, allowing for therapeutic intervention or clinical monitoring before the onset of motor symptoms. This approach, entirely based on rs-fMRI signals, avoids any feature engineering, and demonstrates that it is possible to extract relevant latent signals from raw data alone, as also highlighted by Ryali et al.^16^ in the context of gender differences.

The classification between iRBD and PD proved to be particularly challenging for the model, with only modest balanced accuracy scores (52–60%) even when using transfer learning. This outcome suggests a substantial overlap in the functional patterns of the two conditions, reinforcing the idea of a neurophysiological continuum between iRBD and PD^2,27^. The analysis of the most relevant brain regions revealed a marked involvement of occipital and parietal areas, particularly the bilateral lingual gyri, cuneus and precuneus, while frontal cortical involvement was less evident compared to previous tasks. Notably, these findings are consistent with evidence from FDG-PET studies showing that reduced medial occipital glucose uptake in iRBD is associated with an increased risk of phenoconversion to PD or dementia with Lewy bodies^29^. Although these results should be interpreted with caution due to the limited accuracy of the model, they support the notion that the functional alterations between iRBD and PD are more similar than distinct, further reinforcing the concept of iRBD as an early-stage of Parkinsonian neurodegeneration.

In the present study, specific strategies were adopted to address the complexity and heterogeneity of rs-fMRI signals, one of the main methodological challenges when working with functional brain data, especially in multicenter clinical settings. As also highlighted by Ryali et al.^16^, rs-fMRI signals are notoriously non-stationary and highly variable, not only across individuals but also across sessions from the same subject and across different acquisition protocols. Such variability can compromise analysis quality and hinder the training of robust predictive models. To mitigate these issues, the study implemented a temporal harmonization system across datasets by interpolating the repetition time (TR) of the different cohorts. By standardizing the TR to a common value (1.567 s), it was possible to reduce the impact of technical differences without sacrificing temporal resolution. In addition, subject-specific z-score normalization of the time series was applied to stabilize BOLD signal amplitude, removing spurious variations due to noise or experimental conditions. A key role was also the application of a sliding window approach, which allowed for the generation of multiple temporal segments from each fMRI time series^30^. This strategy, already adopted in recent deep learning studies in neuroimaging, improved the model’s generalization ability and strengthened classification performance by fully leveraging the dynamic component of the signal without introducing artifacts^31,32^.

Several limitations must be considered when interpreting the results of this study. One of the primary limitations is the variability in data acquisition protocols across the different centers involved in this study. For instance, in the PPMI cohort, participants were instructed to keep their eyes open during rs-fMRI acquisition, whereas in the other cohorts, participants were asked to close their eyes. This difference in instructions could influence brain activity, potentially affecting the rs-fMRI measurements. As already discussed, one of the key differences was in the TR, which varied significantly across centers. This disparity in TR values could impact the temporal resolution of the fMRI data and the accuracy with which dynamic brain activity is captured. To address this, an interpolation procedure was applied to standardize the data, but it is important to acknowledge that these differences in acquisition protocols and scanner hardware still introduce variability that could affect the model’s performance.

A second limitation is the data imbalance across centers, particularly where the number of healthy controls is significantly lower than the number of PD patients. This imbalance introduces potential bias, especially affecting the model’s sensitivity to the underrepresented class, which could compromise overall performance. To address this issue, higher weights are assigned to the underrepresented classes and lower weights to the more frequent ones. The purpose of this approach is to ensure the model pays more attention to the underrepresented classes during training, preventing the model from neglecting them and ultimately improving performance on imbalanced datasets.

A further limitation is that the explainability analysis was limited to correctly classified windows, aiming to identify brain regions consistently contributing to successful model decisions while minimizing noisy attribution patterns from misclassified samples. As such, the attribution maps should be interpreted as group-level class fingerprints, rather than comprehensive explanations of all individual predictions.

Moreover, attribution scores were summarized using absolute integrated gradient values, which quantify the overall magnitude of regional relevance but do not preserve the direction of each region’s contribution. This choice was made to obtain a stable group-level measure of regional importance, as averaging signed attributions across time could lead to cancellation effects and underestimation of consistently relevant regions.

The stDNN model developed in this study represents a significant step forward in using rs-fMRI to analyze brain functional connectivity and its application to the early diagnosis and monitoring of neurodegenerative diseases. The model demonstrated high classification accuracy, especially when distinguishing healthy controls from middle-stage PD patients, as well as from iRBD, providing meaningful insights into brain features underlying these conditions. The inclusion of an XAI approach further enhanced the interpretability of the model, making its findings more accessible and useful for clinical applications. The exploration of rs-fMRI as a biomarker for tracking iRBD to PD progression opens new avenues for early diagnosis. Future work will focus on refining the model for higher accuracy in distinguishing iRBD from PD and investigating the longitudinal predictive power of rs-fMRI in neurodegenerative disease progression.

## Methods

### Study population

The study population included patients with idiopathic PD, iRBD, and healthy controls who underwent a baseline brain MRI, including high-resolution T1-weighted imaging and rs-fMRI scans. Participants in this study were selected from three cohorts, each with its own inclusion and exclusion criteria and study protocols (see Supplementary materials for details).

The first was a prospective cohort of iRBD and PD patients recruited at IRCCS Ospedale San Raffaele (OSR) [OSR]. Eighty-six PD patients were recruited at the Neurology Unit of OSR, while 44 patients with iRBD were consecutively recruited at the Sleep Disorders Center, Division of Neuroscience, OSR (Table 2). A group of 129 age- and sex-matched healthy controls was recruited among spouses of patients and by word of mouth (Table 1). All subjects underwent comprehensive neurological, cognitive, and motor functional evaluations, along with a 3T Philips Medical Systems MRI scan (see Supplementary materials for details).

**Table 2:** Binary classification results on testing datasets

	Accuracy	Balanced accuracy	F1 score	Sensitivity	Specificity	Precision
Controls vs PD						
stDNN model *OSR* + *PPMI+Naples*	75.6% ± 1.2	71.0% ± 2.4	82.3% ± 0.8	84.1% ± 2.5	57.8% ± 6.5	80.6% ± 2.0
LSTM model	58.9% ± 6.6	49.8% ± 4.2	70.8% ± 7.3	75.6% ± 14.1	23.9% ± 13.2	67.3% ± 2.2
**Controls vs middle-stage PD**						
stDNN model *OSR* + *PPMI+Naples* *Median UPDRS-III* = *24*	71.9% ± 4.8	71.9% ± 4.8	72.8% ± 4.0	75.0% ±2.6	68.8% ± 8.8	71.0% ± 6.6
**Controls vs early-stage PD**						
stDNN model *OSR* + *PPMI+Naples* *Median UPDRS-III* = *24*	72.8% ± 4.6	72.9% ±4.6	71.9% ± 5.1	70.4% ± 8.0	75.2% ± 3.5	73.6% ± 3.4
**Early-stage PD vs middle-stage PD**						
stDNN model *OSR* + *PPMI+Naples* *Median UPDRS-III* = *24*	68.1% ± 4.4	68.3% ± 4.4	68.1% ± 5.0	69.3% ± 7.3	67.4% ± 7.0	67.2% ± 4.6
**Controls vs iRBD**						
stDNN model *OSR* + *PPMI*	71.3% ± 3.7	71.1% ± 3.6	68.8% ± 3.4	68.1% ± 4.8	74.1% ± 6.0	69.7% ± 4.3
stDNN model + Transfer learning*OSR* + *PPMI*	82.6% ± 3.6	82.1% ± 3.6	80.0% ± 4.1	75.0% ± 5.0	89.1% ± 4.1	85.9% ± 4.9
**iRBD vs PD**						
stDNN model *OSR* + *PPMI*	60.6% ± 8.9	52.0% ± 3.3	71.0% ± 11.0	73.5% ± 21.2	30.5% ± 21.7	71.0% ± 1.9
stDNN model + Transfer learning *OSR* + *PPMI*	66.5% ± 3.8	59.1% ± 4.1	76.4% ± 3.0	77.8% ± 4.0	40.3% ± 6.7	75.2% ± 2.7
**iRBD vs middle-stage PD**						
stDNN model *OSR* + *PPMI* *Median UPDRS-III* = *24*	56.8% ± 5.2	56.5% ± 5.0	59.9% ± 6.2	59.2% ± 9.0	53.1% ± 3.9	60.2% ± 4.1

Values are presented as the mean ± standard deviation of the evaluation metric applied to the test set on each task, calculated across cross-validation folds.

*iRBD* isolated REM sleep behavior disorder, *PD* Parkinson’s disease, *stDNN* spatiotemporal deep neural network.

The second cohort included iRBD and PD patients from PPMI database [PPMI]. The PPMI clinical study is a long-term, observational research project aimed at understanding the progression of PD through clinical features and different imaging, biological, and genetic markers. For up-to-date information, see www.ppmi-info.org/. For the current study, we analyzed a sample of 247 PD patients, 100 iRBD patients, and 37 healthy controls (Table 1). Details about the PPMI MRI data acquisition protocol can be seen on the official PPMI webpage.

The third was a prospective cohort of de novo PD patients recruited at University of Campania “Luigi Vanvitelli” in Naples [Naples]. Ninety de novo PD patients were consecutively recruited at the time of clinical diagnosis at the Movement Disorders Unit, First Division of Neurology, University of Campania “Luigi Vanvitelli” in Naples, Italy^21^. All patients underwent cognitive and clinical assessments along with MRI scans at study entry. Thirty-eight age- and sex- matched controls were also recruited (Table 1). Baseline 3DT1-weighted and rs-fMRI images were acquired on the General Electric (GE) 3 Tesla MRI scanner. Detailed description of inclusion/exclusion criteria, clinical and cognitive evaluations, and MRI protocol are reported in Supplementary materials.

For all the cohorts involved, approval was received from the local ethical standards committees (IRCCS Ospedale San Raffaele, Milan, Italy for the OSR cohort; www.ppmi-info.org/ for the open access PPMI database cohort; University of Campania “Luigi Vanvitelli”, Naples, Italy for the Naples cohort) on human experimentation and written informed consent was obtained from all subjects prior to study participation. The study was conducted in accordance with the Declaration of Helsinki and its subsequent amendments.

### RS fMRI data pre-processing

3D T1-weighted and rs-fMRI pre-processing are described in detail in the Supplementary materials. For each subject, structural (3D T1-weighted) and rs-fMRI data were processed to parcellate the brain into 220 similarly sized gray matter regions belonging to the automated anatomical labeling (AAL) atlas, as previously described^33^. The resulting ROIs were registered into each subject’s functional space using both linear (FLIRT) and non-linear (FNIRT) transformations. After preprocessing the rs-fMRI data, the mean BOLD time series were extracted from each ROI, capturing the average temporal dynamics of neural activity across the scan.

Due to variations in acquisition protocols across cohorts, different time repetition (TR) intervals were used for rs-fMRI scans. To address this issue, an interpolation was implemented to resample the time series data to a common TR while preserving the underlying characteristics of the original signal. The interpolation process was implemented using the *interp1d* function from the *SciPy* library^34^ in Python, utilizing a cubic spline interpolation. Timeseries extracted from subjects in the OSR (TR = 1.567 and TR = 3.0), PPMI (TR = 2.5 and TR = 2.4), and Naples (TR = 1.508) datasets were interpolated to a TR of 1.567 s, corresponding to the TR of the OSR dataset. This TR was selected for an optimal balance between minimizing the number of subjects requiring interpolation, since it was the second most common TR across datasets, and maintaining high temporal resolution, given that it was among the shortest available TRs, thus preserving a good quantity of temporal information on the BOLD signal fluctuations.

After the initial preprocessing, the time series data were organized into a 3D array structure. Each subject was represented by a 2D matrix with dimensions corresponding to the number of ROIs of the atlas (NC = 220) and the number of time points (NT) (Fig. 2.1). The first 10 time points were removed from each time series, as they may not accurately reflect the resting state of the subjects.

**Fig. 2:**
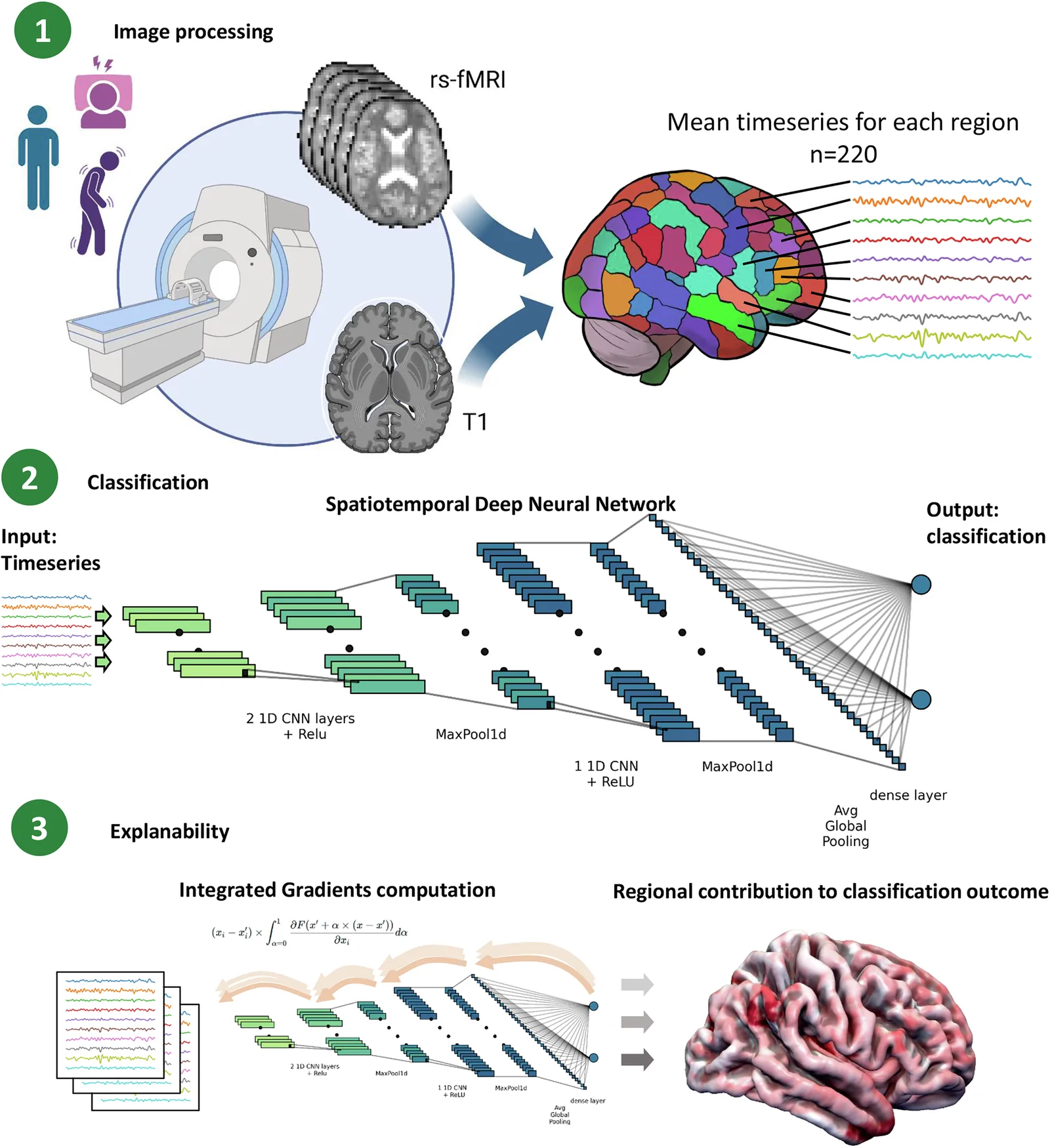
Schematic overview of the explainability framework for resting-state functional MRI-based classification and brain fingerprint extraction. 1) Image processing: resting state functional MRI and 3DT1-weighted images are preprocessed and parcellated, and the mean BOLD time series is extracted for each brain region (*n* = 220). 2) Classification: regional time series are provided as input to a spatiotemporal deep neural network producing a binary prediction distinguishing PD/iRBD from controls and between each other. 3) Explainability: Integrated Gradients are computed with respect to the network input and summarized as regional contribution weights, yielding individualized brain fingerprints and group-level fingerprints obtained by averaging across participants within each class. CNN convolutional neural network, rs fMRI resting-state functional MRI.

To further standardize the data, Z-score normalization was applied to the time series for every subject, preserving the relative amplitude of timeseries within the individual subject.

### Spatiotemporal deep neural network architecture

The stDNN model was implemented using the *Pytorch* library in Python. The architecture consists of three primary processing stages: feature extraction using one-dimensional convolutional layers, followed by features aggregation through temporal averaging operation, and classification via a sigmoid layer (Fig. 2.2).

In the first block, the model employs two convolutional layers with 256 filters. The input to this block is a tensor with a dimensionality of NC×NT, where NC = 220 represents the number of ROIs for AAL atlas while NT is the length of time windows (NT = 117 time points). The 1D convolutional layers utilize a kernel size of 7, this transformation yields a spatiotemporal representation of size 256 × NT. Following this, a Rectified Linear Unit (ReLU) activation function is applied. To further enhance feature extraction and reduce the temporal dimension of the data, a maxpool layer with a kernel size of 4 and a stride of 2 is applied after each convolutional block.

The second block of the stDNN consists of a single 1D convolutional layer with 512 filters and a smaller kernel size of 5, followed by ReLU activation and another maxpool layer. The second layer produces a representation of size 512 × NT.

Instead of using a traditional flattening operation, the stDNN model incorporates a “temporal averaging layer”, calculating the average value of the features extracted by each filter over the entire temporal dimension. The final output of the temporal averaging layer reduces the original sequence to a single value per channel, resulting in a final output of 512 values. To address potential overfitting, a dropout layer is integrated before the final layer with a dropout rate of 0.5. The final step is a fully connected layer which outputs 2 values, followed by a sigmoid activation function for binary classification (see Supplementary materials for details).

### Train-validation-test partitioning and five-fold cross-validation

For each classification task, the dataset model performance was evaluated using a subject-wise five-fold cross-validation procedure. At each iteration, one-fold was used as the test set, while the remaining subjects were used for model development. Within the development set, separate training and validation partitions were defined. Therefore, the train-validation-test split refers to the partitioning performed within each cross-validation iteration, rather than to a single fixed split applied once to the entire dataset. This procedure was repeated across all five folds, so that each subject served once as test data. Final performance metrics were computed on the held-out test set of each fold and then averaged across folds. The validation set was employed to monitor the minimization of the loss function on previously unseen data during the training process, thereby mitigating the risk of overfitting.

A dual-layer stratification approach was implemented by considering both class information (such as healthy controls, iRBD and PD) and the center of origin, maintaining the same proportion of different classes and centers of origin in the train, test and validation sets.

### stDNN *comparisons*

The proposed model was evaluated across multiple binary classification tasks to assess its performance in distinguishing between different subject groups.

*Controls vs PD*. The primary classification task involved differentiating individuals with PD from healthy controls using the complete dataset. As a benchmark, the same classification was performed using a LSTM network, a state-of-the-art architecture for time series classification (more details regarding this architecture in the Supplementary materials).

To further investigate the model’s ability to discriminate between different stages of PD, subjects were stratified based on the median UPDRS-III score (24) Three additional classification tasks were conducted: controls *vs* early-stage PD patients, controls *vs* middle-stage PD patients, early-stage *vs* middle-stage PD patients. In addition, alternative classification contrasts were explored by stratifying subjects according to different UPDRS-III thresholds, including both the median and the third quartile of the score distribution, in order to assess the robustness of the model across varying disease severity definitions.

*Controls vs iRBD*. Binary classification was performed between individuals with iRBD and controls from the OSR and PPMI cohorts, as the Naples dataset includes only PD and control subjects. A transfer learning approach was employed, initializing the model with weights from the controls *vs* PD classification.

*iRBD vs PD*. In order to distinguish iRBD from PD, classification was performed using subjects from the PPMI and OSR cohorts, as the Naples dataset does not include iRBD cases. A transfer learning approach was applied, leveraging the pre-trained PD *vs* controls model. Additionally, the model’s performance in differentiating iRBD from middle-stage PD was evaluated by selecting PD patients with UPDRS-III scores above the median (24). In addition, alternative classification contrasts were explored by stratifying subjects according to different UPDRS-III thresholds, including both the median and the third quartile of the score distribution, in order to assess the robustness of the model across varying disease severity definitions.

### Sliding-window segmentation

The time series were subdivided into smaller windows to effectively enhance the dataset. A similar approach was implemented by Zhao et al.^35^, who utilized the sliding-window method in rs-fMRI data, considering the window size as a crucial factor in determining classification accuracy. In the present implementation, the window size was initially set to 117 time points (equivalent to 184 s), with the distance between consecutive windows determined by a step size of 25 time points (40 s), based on the parameters used in the paper by Ryali et al.^16^. Additional tests were performed by varying these parameters, but the changes resulted in a decrease in performance, leading to the retention of the initial parameters. Importantly, data partitioning was performed at the subject level before window generation. Within each cross-validation fold, each subject was assigned exclusively to one partition (training, validation, or test), and the sliding-window procedure was subsequently applied independently within that partition only. Therefore, all windows derived from a given subject remained in the same partition, and no windows from the same subject were shared across training, validation, and test sets, preventing any potential data leakage.

Using this procedure, each subject’s time series was segmented into five overlapping windows. During inference, predictions were first generated at the window level and then aggregated at the subject level. The final predicted label for each subject in the test set was determined by majority voting across the five windows derived from that subject.

### Evaluation metrics

Performance metrics were calculated on the held-out test set at each cross-validation iteration, and final results were reported as mean ± standard deviation across the five test folds. To evaluate the performance of the model, several metrics were used to provide a comprehensive assessment of its ability to correctly classify subjects, while accounting for class imbalance: Sensitivity (Recall), Precision, Specificity, Accuracy, Balanced Accuracy, and F1 Score.

### Transfer learning

A transfer learning approach was employed. Specifically, a model pre-trained on the classification of healthy controls relative to PD patients was adapted for the more complex tasks of this work, where classification boundaries may be less apparent. The model was then fine-tuned for the current task, freezing the weights for the convolutional layers, and only the fully connected layer was retrained on the new classification task.

### Model explainability

To identify which brain regions were the most influential in the classification, we employed integrated gradients, a feature attribution method that quantifies the contribution of each input feature to the model’s predictions (Fig. 2.3).

In the current analysis, the results of the integrated gradients were computed for individual temporal windows across the fMRI data utilizing the *Captum* library in Python. Only the windows that were correctly classified for each subject were provided as input to the model, in order to analyze which brain areas were most involved in the classification process when it led to correct predictions. To ensure a comprehensive evaluation of each window’s influence on classification, the absolute values of the integrated gradients were averaged. This approach accounted for both positive and negative contributions, allowing for a balanced representation and more easily interpretation of each window’s effect on the model’s output. Subsequently, for each ROI across all subjects, mean integrated gradient values were computed and plotted on a brain template to enhance visual interpretability.

## Supplementary information

Supplementary materials R1.

## Data Availability

The datasets generated and/or analyzed during the current study are not publicly available due to ethical and privacy restrictions related to the use of clinical data from human participants, but anonymized data are available from the corresponding author on reasonable request and subject to institutional approval.

## Code availability

The code is available from the corresponding author on reasonable request.
