# Supplementary material for: Spatiotemporal deep learning for early detection of isolated REM sleep behavior disorder and Parkinson’s disease using functional MRI data
Source: NPJ Parkinsons Dis. 2026 Jul 14;12:273. doi: 10.1038/s41531-026-01477-7 (PMC13737112; doi:10.1038/s41531-026-01477-7)
Supplement: Supplementary file 1 [file 41531_2026_1477_MOESM1_ESM.pdf]

## SUPPLEMENTARY MATERIALS

### Inclusion/exclusion criteria

### Prospective cohort of RBD and PD patients [OSR]

Inclusion criteria for PD participants were a diagnosis of PD according to current diagnostic criteria (1) with Hoehn and Yahr scale (H&Y) score ≤ 3.

Inclusion criteria for patients and healthy controls were: i) right handedness; ii) native Italian speakers; iii) age 50-75 years; iv) Mini Mental State Examination (MMSE) score ≥ 24; v) oral and written informed consent to study participation.

Inclusion criteria for the control group were: normal neurological assessment, MMSE score ≥26, no family history of neurodegenerative diseases.

Exclusion criteria for both patients and healthy controls included: the presence of medical illnesses or substance abuse that could interfere with cognitive function; any major systemic, psychiatric, neurological, or musculoskeletal disease; other causes of gait impairment (e.g., rheumatic, orthopedic, or musculoskeletal conditions); history of alcohol and/or psychotropic drug abuse; secondary forms of RBD or PD as determined by historical data or neurological examinations; contraindications to MRI (such as cardiac pacemakers, ferromagnetic foreign bodies, or claustrophobia); evidence of brain damage on routine MRI, including lacunar infarcts or extensive cerebrovascular disorders.

### RBD and PD patients from PPMI database [PPMI]

Patients were invited to the PPMI study if they were of at least 30 years of age and were selected for study participation if they fulfilled four criteria: 1. presented two of three cardinal symptoms (bradykinesia, rigidity or resting tremor); 2. diagnosis within 2 years prior to entering the study; 3. untreated for PD on entering the study; 4. had a deficit of dopamine transporter, as assessed by

123I-ioflupane dopamine transporter Datscan imaging.(2) More details about the PPMI inclusion and exclusion criteria can be seen on the official PPMI webpage (see www.ppmi-info.org/).

**Prospective cohort of de novo PD patients [Naples]**

Patients with PD were included according to clinical diagnosis of PD based on the modified diagnostic criteria of the UK Parkinson's Disease Society Brain Bank (3). Only subjects with a modified Hoehn and Yahr (mH&Y) stage of 2.5 or lower were considered eligible for inclusion. Exclusion criteria included PD onset before the age of 40, any prior treatment with dopaminergic, anticholinergic, antidepressant, or other centrally acting medications, significant cognitive impairment attributable to PD, or any other clinically significant medical condition.

**Clinical evaluation**

**Prospective cohort of iRBD and PD patients [OSR]**

An experienced neurologist blinded to MRI results performed clinical assessments. PD patients were examined in ON state (i.e., period when the dopaminergic medication is working, and symptoms are well controlled). Demographic, general clinical and family data (sex, education, age, handedness, age at onset, side of onset, PD duration and family history) were obtained using a semi-structured interview. Levodopa equivalent daily dose (LEDD) was calculated. Disease severity was defined using the H&Y stage score and Unified Parkinson's Disease Rating Scale (UPDRS). UPDRS was used to evaluate non-motor symptoms (UPDRS I), motor symptoms (UPDRS II), and motor signs (UPDRS III). Moreover, an experienced neurologist assessed sleep quality and motor/non-motor impairments in iRBD patients with the MDS-UPDRS (4) and the Non-Motor Symptoms Scale (NMS) (5).

Patients and healthy controls underwent a comprehensive neuropsychological assessment covering several cognitive domains.

**RBD and PD patients from PPMI database [PPMI]**

Clinical assessments of motor and non-motor characteristics comprised the MDS-UPDRS, including modified Hoehn and Yahr stage, ESS and RBDSQ were extracted from the PPMI dataset.

**Prospective cohort of de novo PD patients [Naples]**

Motor assessment was performed in the "practical off" state (when the patient was free of dopaminergic medication overnight for a period of at least 12 hours). Disease severity and motor performance were assessed using the H&Y stages and the UPDRS III. Global cognitive functioning was assessed in patients and controls. For details concerning neurophyscological assessment see De Micco et al. (6)

**MRI acquisition protocols**

**Prospective cohort of iRBD and PD patients [OSR]**

Patients and healthy controls underwent brain MRI using a 3T scanner (Philips Medical Systems, Best, the Netherlands) at San Raffaele Hospital. The imaging protocol included various sequences to ensure comprehensive evaluation and exclusion of any structural brain alterations or excessive vascular lesions. The first sequence was a 3D T1-weighted turbo field echo (TFE) with a TR of 7 ms, TE of 3.2 ms, and TI of 1000 ms, resulting in 204 sagittal slices with a voxel size of 1 × 1 × 1 mm, a matrix of 256 × 256, and a field of view (FOV) of 256 × 256 mm². Additionally, T2*-weighted single-shot echo-planar imaging (EPI) was utilized for rs-fMRI), with a TR of 1567 ms, TE of 35 ms, and a flip angle of 70°, covering 320 sets of 48 axial slices with a thickness of 3 mm and a FOV of 240 mm², with a pixel size of 2.5 × 2.5 mm.

Furthermore, another group of OSR subjects underwent MRI using a 3.0T Philips Intera scanner (Best, the Netherlands). This older dataset, referred to as *OSR_cohort1*, features different acquisition characteristics. MRI analysis in this context involved T2*-weighted single-shot echo-planar images for resting-state fMRI with a TR of 3000 ms, TE of 35 ms, and a flip angle of 90°. The

protocol included 200 sets of 30 contiguous axial slices, each 4 mm thick, with a field of view of 240 $mm^2$ and a matrix size of 128 × 128. Additionally, other sequences of interest from this scan protocol included a 3D T1-weighted fast field echo with TR of 25 ms, TE of 4.6 ms, a flip angle of 30°, 220 contiguous axial slices with a FOV of 230 × 182 $mm^2$ and a voxel size of 0.89 × 0.89 × 0.8 mm.

During the resting-state fMRI scans, subjects were also instructed to remain motionless, keep their eyes closed, and avoid thinking about anything specific. Fat saturation was applied to minimize chemical shift artifacts.

**RBD and PD patients from PPMI database [PPMI]**

A high-resolution T1-weighted, 3D volumetric sequence has been acquired in the sagittal plane with a slice thickness of 1.0 mm, consisting of 192 slices, with a voxel size of 1.0 mm × 1.0 mm in-plane resolution, and an anterior-posterior (AP) phase encoding direction. The matrix size is 256 × 256, with no interpolation, zero-filling, or ZIP factor permitted.

A total of 341 volumes of rs-fMRI were acquired. The first sequence was configured as an axial oblique sequence, aligned parallel to the AC-PC line. It featured a slice thickness of 3.5 mm with no gap, encompassing approximately 40 slices. The phase encoding direction is from right to left (R >> L), and the matrix size is 64 × 64. The FOV measures 224 mm × 224 mm, with a TR of 2500 ms, a TE of 30 ms, and a flip angle of 80 degrees. Slices are acquired in an interleaved order, resulting in a total of 240 measurements collected over approximately 10 minutes, utilizing in-plane acceleration via GRAPPA or SENSE (factor of 2). Participants were instructed to keep their eyes open and remain still during the scan.

The remaining 43 volumes were obtained using a 3T MRI system with a flip angle of 80 degrees, produced by Siemens. This sequence had a matrix size of 476x462, with a voxel size of 3.29x3.29 mm and a slice thickness of 3.30 mm. The pulse sequence employed was echo planar and the scan comprised 210 volumes, with a TR of 2400 ms and a TE of 25 ms.

**Prospective cohort of de novo PD patients [Naples]**

MR images were acquired on a General Electric 3 Tesla MRI scanner equipped with an 8-channel parallel head coil at the MRI Research Center, University of Campania "Luigi Vanvitelli" Napoli. Three-dimensional high-resolution T1-weighted sagittal images (IR-FSPGR sequence, TR = 6988 ms, TI = 1100 ms, TE = 3.9 ms, flip angle = 10, voxel size = 1 x 1 x 1.2 $mm^3$) were acquired. fMRI data consisted of 240 volumes of a repeated gradient-echo echo planar imaging T2*-weighted sequence (TR = 1508 ms, axial slices = 29, matrix = 64 x 64, field of view = 256 $mm^2$, thickness = 4 mm, interslice gap = 0 mm). During the fMRI scan, subjects were asked to simply stay motionless, awake, with their eyes closed.

**MRI Analysis pipeline**

**T1 processing**

Grey matter (GM) was parcellated using an innovative approach that delineated 220 similarly sized regions, effectively balancing the need for a substantial number of equal-sized nodes while respecting anatomical landmarks (7). The process began with the automated anatomical labeling (AAL) atlas, which divides the brain into 116 ROIs. A K-means algorithm was employed to optimize the number of ROIs, ultimately resulting in 262 regions, identified by the lowest ratio of the interquartile range to the median ROI volume. After dividing the 116 AAL ROIs into 262 approximately equal-sized partitions, 42 cerebellar ROIs were excluded, yielding a final total of 220 GM regions for analysis. To integrate the 220 ROIs into each subject's space, a series of registrations were employed to ensure accurate alignment and mapping of the GM regions. This process of spatial normalization, involved calculating and concatenating the transformations between the subject's T1-weighted image and the MNI152 standard space using both linear and non-linear methods, implemented through FLIRT (8) and FNIRT in FSL.

**fMRI processing**

Resting-state fMRI data were pre-processed using the Statistical Parametric Mapping software package (SPM12) in conjunction with Matlab and FSL. Before conducting statistical analyses, all images underwent a series of preprocessing steps aimed at minimizing the influence of data acquisition and physiological artifacts to achieve increased validity and sensitivity in group analysis. Initially, motion correction was performed and images were realigned to the first volume by the FSL tool MCFLIRT; next, linear detrending and band-pass filtering (0.01–0.08 Hz) were performed using the REST software to eliminate low-frequency drifts and high-frequency physiological noise. Furthermore, non-neuronal sources of synchrony and motion-related artifacts were minimized by regressing out six motion parameters estimated by MCFLIRT, along with average signals from the ventricular cerebrospinal fluid (CSF) and white matter (WM). This was followed by spatial smoothing with a Gaussian kernel of full width at half maximum (FWHM) of 6 mm. Finally, slice-timing correction was performed, and the images were co-registered to the subject’s 3D T1-weighted image in order to map the functional data onto a high-resolution structural MRI.

**Mean timeseries extraction**

From each ROI mean time series were extracted by averaging the signal from all corresponding voxels inside each region according to the 220 ROIs of the AAL k-means atlas. The rs-fMRI data were masked with the subjects' GM and basal ganglia maps to include only voxels related to these structures. Cortical GM was segmented using SPM12, while maps of the basal ganglia (including the bilateral caudate, globus pallidus, putamen, and thalamus), hippocampus, and amygdala were obtained using FIRST (9) in FSL.

**Spatiotemporal Deep Neural Network architecture**

Class imbalance was addressed by calculating class weights using the compute_class_weight function from the scikit-learn utils module. These computed weights are then passed to the cross-

entropy loss function, which adjusts the loss and gradient calculations according to the relative frequency of each class. The optimizer selected for training is *AdamW*, which combines the benefits of the *Adam* optimizer with weight decay for improved generalization.

Hyperparameters for the deep learning model training were manually tuned across different experiments to optimize performance for specific classification tasks. Parameters such as learning rate, batch size, weight decay and number of epochs were iteratively adjusted based on preliminary results, aiming to maximize balanced accuracy, starting with the following initial values: learning rate = 0.000001, batch size = 8, weight decay = 0.0001 and number of epochs = 150.

The training process incorporates a learning rate scheduler that monitors the validation loss and reduces the learning rate by a factor of 0.05 if no improvement is observed over a number of 10 epochs promoting better convergence toward optimal performances.

**REFERENCES**


1. R. B. Postuma *et al.*, MDS clinical diagnostic criteria for Parkinson's disease. *Mov Disord* **30**, 1591-1601 (2015).
2. M. A. Nalls *et al.*, Diagnosis of Parkinson's disease on the basis of clinical and genetic classification: a population-based modelling study. *Lancet Neurol* **14**, 1002-1009 (2015).
3. W. R. Gibb, A. J. Lees, The relevance of the Lewy body to the pathogenesis of idiopathic Parkinson's disease. *J Neurol Neurosurg Psychiatry* **51**, 745-752 (1988).
4. C. G. Goetz *et al.*, Movement Disorder Society-sponsored revision of the Unified Parkinson's Disease Rating Scale (MDS-UPDRS): scale presentation and clinimetric testing results. *Mov Disord* **23**, 2129-2170 (2008).
5. K. R. Chaudhuri *et al.*, The metric properties of a novel non-motor symptoms scale for Parkinson's disease: Results from an international pilot study. *Mov Disord* **22**, 1901-1911 (2007).
6. R. De Micco *et al.*, Functional Connectomics and Disease Progression in Drug-Naive Parkinson's Disease Patients. *Mov Disord* **36**, 1603-1616 (2021).
7. M. Filippi *et al.*, Brain network connectivity differs in early-onset neurodegenerative dementia. *Neurology* **89**, 1764-1772 (2017).
8. M. Jenkinson, P. Bannister, M. Brady, S. Smith, Improved optimization for the robust and accurate linear registration and motion correction of brain images. *Neuroimage* **17**, 825-841 (2002).
9. B. Patenaude, S. M. Smith, D. N. Kennedy, M. Jenkinson, A Bayesian model of shape and appearance for subcortical brain segmentation. *Neuroimage* **56**, 907-922 (2011).

**Supplementary Table 1.** Binary classification results on testing datasets.

| | Accuracy | Balanced accuracy | F1 score | Sensitivity | Specificity | Precision |
|---|---|---|---|---|---|---|
| **Controls vs PD** | | | | | | |
| **stDNN model**<br>***OSR+PPMI*** | 78.0% ± 3.4 | 74.9% ±3.9 | 83.6% ±2.6% | 84.1% ±2.8 | 65.7% ±6.1 | 83.1% ±2.9 |
| **Controls vs middle-stage PD** | | | | | | |
| **stDNN model**<br>***OSR+PPMI+Naples***<br>***3rd quartile UPDRS-III= 33.25*** | 58.9%±7.7 | 58.9%±7.7 | 47.3%±23.6 | 43.3%±25.9 | 74.4%±12.8 | 58.3%±14.5 |
| **stDNN model**<br>***OSR+PPMI***<br>***Median UPDRS-III= 26*** | 65.9% ±6.3 | 66.0% ±6.3 | 65.6% ±5.9 | 64.5% ±6.0 | 67.4% ±9.2 | 67.0% ±7.3 |
| **Controls vs early-stage PD** | | | | | | |
| **stDNN model**<br>***OSR+PPMI***<br>***3rd quartile UPDRS-III= 33.25*** | 74.3%±3.1 | 73.7%±2.8 | 77.4%±3.4 | 77.8%±5.7 | 69.6%±3.1 | 77.2%±1.6 |
| **stDNN model**<br>***OSR+PPMI***<br>***Median UPDRS-III= 26*** | 75.9%±4.3 | 75.8%±4.3 | 76.3%±5.6 | 77.5%±9.0 | 74.1%±1.6 | 75.3%±2.4 |
| **Early-stage PD vs middle-stage PD** | | | | | | |
| **stDNN model**<br>***OSR+PPMI*** | 74.2%±1.9 | 49.8%±0.4 | 1.7%±3.9 | 1.1%±2.5 | 98.5%±3.3 | 4.0%±8.9 |

| | | | | | | |
|---|---|---|---|---|---|---|
| ***3rd quartile UPDRS-III= 33.25*** | | | | | | |
| **stDNN model** ***OSR+PPMI*** ***Median UPDRS-III= 26*** | 63.0%±5.3 | 62.7%±5.7 | 58.6%±9.1 | 56.5%±12.9 | 68.9%±5.0 | 61.8%±5.2 |
| iRBD vs middle-stage PD | | | | | | |
| **stDNN model** ***OSR+PPMI*** ***3rd quartile UPDRS-III= 33.25*** ***(RBD = 90)*** | 53.3%±3.0 | 53.3%±3.0 | 54.3%±11.7 | 60.0%±21.3 | 46.7%±25.0 | 55.2%±6.8 |
| **stDNN model** ***OSR+PPMI*** ***3rd quartile UPDRS-III= 33.25*** ***(RBD = 144)*** | 56.4%±7.3 | 51.7%±7.7 | 28.7%±21.7 | 31.1%±33.9 | 72.4%±26.6 | 34.5%±23.0 |
| **stDNN model** ***OSR+PPMI*** ***Median UPDRS-III= 26*** | 58.9%±8.5 | 58.2%±9.2 | 66.6%±2.8 | 79.5%±16.1 | 36.8%±33.3 | 59.5%±8.9 |

Values are presented as the mean ± standard deviation of the evaluation metric applied to the test set on each task, calculated across cross-validation folds. *Abbreviations: iRBD*= isolated REM sleep behavior disorder*; PD= Parkinson's disease;* stDNN= spatiotemporal deep neural network.
